# SINFONIA study protocol: a phase II/III randomised controlled trial examining benefits of guided online group singing in people with chronic obstructive pulmonary disease and interstitial lung disease and their carers

**DOI:** 10.1186/s12931-022-02133-3

**Published:** 2022-08-16

**Authors:** Natasha Smallwood, Amy Pascoe, Sara Vogrin, Jennifer Philip

**Affiliations:** 1 Department of Respiratory Medicine, The Alfred Hospital, 55 Commercial Road, Prahran, VIC 3004 Australia; 2 Department of Allergy, Immunology and Respiratory Medicine, Central Clinical School, The Alfred Hospital, Monash University, Melbourne, VIC 3004 Australia; 3 Department of Medicine, St Vincents Hospital, University of Melbourne, Fitzroy, VIC 3065 Australia; 4 Faculty of Medicine, Dentistry, and Health Sciences, University of Melbourne, Melbourne, VIC Australia; 5 Centre for Palliative Care, St Vincent’s Hospital, Fitzroy, Australia

**Keywords:** Chronic obstructive pulmonary disease, Interstitial lung disease, Advanced lung disease, Breathlessness, Community, Intervention, Singing, Music therapy, Online

## Abstract

**Background:**

Chronic obstructive pulmonary disease (COPD) and interstital lung disease (ILD) are incurable conditions characterised by airflow limitation, persisting respiratory symptoms, and progressive respiratory failure. People living with COPD or ILD often suffer from chronic and severe breathlessness, with limited treatment options and low engagement rates with current therapies. Group singing represents a potential community-based therapy to improve quality of life for patients with COPD or ILD and breathlessness.

**Methods:**

This protocol papers describes SINFONIA, a parallel, double-arm, randomised, blinded-analysis, mixed-methods phase II/III trial of guided, online group singing that will be conducted over 24 months. Adults with confirmed COPD or ILD, on stable treatment for at least four weeks at time of recruitment, with a modified Medical Research Council (mMRC) dyspnoea score of two or greater, who are capable and willing to give consent, and not currently participating in pulmonary rehabilitation will be eligible to participate. Carers may optionally enrol in the trial. Data will be collected on quality of life, anxiety and depression, breathlessness, mastery of breathing, exercise tolerance, loneliness, healthcare utilisation, and carer quality of life (optional). Participants will be randomised 1:1 to intervention or control arms with intervention arm attending one 90 min, guided, online, group singing session per week for 12 weeks and control arm continuing routine care. Phase II of the trial aims to determine the feasibility and acceptability of guided, online group singing and will collect preliminary data on effectiveness. Phase III aims to determine whether guided, online group singing has an effect on quality of life with the primary outcome being a between arm difference in quality of life (36-item Short Form Survey) measured at 12 weeks.

**Discussion:**

SINFONIA is the first study is the first of its kind in Australia and to our knowledge, the first to deliver the singing intervention program entirely online. Determining the feasibility, acceptability, and effectiveness of guided, online group singing is an important step towards improving low-cost, low-risk, community-based therapeutic options for patients living with COPD or ILD and breathlessness.

*Trial registration:* Phase II—ACTRN12621001274864, registered 20th September 2021; Phase III—ACTRN12621001280897, registered 22nd September 2021.

## Background

Chronic obstructive pulmonary disease (COPD) is an incurable condition characterised by airflow limitation, persisting respiratory symptoms, and progressive respiratory failure [1]. In Australia, COPD represents 43% of all chronic respiratory disease burden [2], affecting 30% of people aged over 75 years [3], with disproportionate impacts on those living in regional areas or with lower socioeconomic status [4]. With over 72,000 admissions each year attributable to COPD, it is the third leading cause of avoidable hospitalisation and generates significant healthcare costs [5, 6]. Internationally, COPD is the second most common respiratory disease after asthma in the United Kingdom [7] and the third leading cause of death worldwide [8]. Interstitial lung disease (ILD) is an umbrella term which captures a large group of diseases resulting in fibrosis of the lungs [9], which often generate distressing, progressive symptoms, and account for a further 8% of chronic respiratory disease burden in Australia [2].

Breathlessness is *“a subjective experience of breathing discomfort that consists of qualitatively distinct sensations that vary in intensity”* which derive from a complex interaction of physiological, psychological, and environmental factors [10]. It is the most common symptom in people with advanced lung disease (ALD), including COPD [11] and ILD [12], and is notoriously difficult to treat [13, 14]. Breathlessness is frequently associated with depression, anxiety, loss of function, social isolation and reduced quality of life [15–17]. Additionally, the presence of breathlessness is a predictor of unscheduled healthcare use, hospitalisation and mortality in patients with ALD [18, 19].

Pulmonary rehabilitation (PR) is a well-established, holistic, therapeutic program encompassing tailored exercise, breath training, psychosocial counselling, and patient education, which has been shown to improve symptoms and function, and reduce hospitalisations [20]. Despite these benefits, referral to, and completion of, PR programs in patients with ALD is alarmingly low [21]. Of the nearly 1.5 million older Australians living with symptomatic COPD, fewer than 10% have ever accessed a program [22]; and internationally fewer than 3% have accessed pulmonary rehabilitation after hospitalisation for an exacerbation of their disease [23]. Participation rates for people with ILD are not well documented. Common barriers to participating in PR include: poor understanding of the program by health professionals and patients, complicated referral pathways, challenges travelling long distances or attending in person (due to symptoms or lack of social support) [24], and limited availaibility of in-person programs in many areas (which has been exacerbated by the COVID-19 pandemic) [23]. Given these challenges, there is a need for sustainable, novel, community-based interventions that are acceptable to patients and their carers, and effective in reducing symptom burden.

Singing is a low-cost, low-risk activity that is recommended for a range of health conditions including neurological [25], mental health [26], and respiratory disorders [27]. Singing can be delivered as a guided, weekly, group-based activity, emphasising focus and control of breathing for patients with ALD. Trials of guided, group singing as a respiratory health intervention have been conducted in New Zealand [28], China [29], Brazil [30], and the United Kingdom [31–33]. Although evidence of robust physiological benefits is mixed [30, 34, 35], studies report improvements to anxiety and depression [28, 29, 33, 35], exercise tolerance [28], and overall quality of life [29–32, 35]. These trials are frequently limited by low study numbers and high risk of bias [27, 36]. To date there are no trials of singing as a therapeutic intervention for ALD in Australia, or in patients with ILD internationally. Robust and long-term studies in the Australian context and in different disease groups are necessary to determine the acceptability and healthcare cost–benefit of guided, group singing for patients with ALD.

Restrictions imposed by the COVID-19 pandemic limit the feasibility of face-to-face singing groups given the aerosol generating nature of singing, and raise the question of whether guided, group singing can offer the same benefits when the intervention is delivered entirely online. Nevertheless, online delivery represents an attractive opportunity to improve healthcare access for participants with limited mobility, poor health, or who live in a rural location with limited access to health services. This is particularly relevant in Australia, where a third of the population lives in regional or remote areas with reduced service availability [37] despite chronic respiratory disease being overrepresented amongst this group [38]. The SINFONIA study (a clinical trial examining the benefits of **S**ing**IN**g **F**or breathing in C**O**PD a**N**d **I**LD p**A**tients) aims to determine the feasibility, acceptability, and effectiveness of guided, online, group singing as an intervention to improve quality of life in patients with COPD or ILD with significant breathlessness living in Australia.

## Methods

### Study design and setting

A parallel, double-arm, randomised, blinded-analysis, mixed-methods phase II/III trial of guided, online group singing will be conducted over 24 months. Adults with COPD or ILD and their adult carers (optional) will be eligible to participate. Methods reported in this paper are reflective of protocol version 1.2, 30^th^ September 2021. Participants will be recruited from tertiary respiratory care clinics based at the Alfred Hospital, Austin Health, Royal Melbourne Hospital, and St Vincent’s Hospital in Melbourne, Australia. People who are not cared for by these health services may also be referred to the study by their primary care provider (e.g., general practitioner) or by self-referral. An advertising poster with eligibility criteria and contact details will be circulated amongst specialist and community pulmonary care services to enable referral. All referred patients will be reviewed by an investigator at a study site to confirm eligbility. Ethics approval for the study was obtained from the St Vincent’ Hospital Melbourne Human Research and Ethics Committee (LRR 237/21).

### Participants

Adults with confirmed COPD or ILD, on stable treatment for at least four weeks at time of recruitment, with a modified Medical Research Council (mMRC) dyspnoea score of two or greater, who are capable and willing to give consent, are eligible to participate. Diagnosis of COPD or ILD must be confirmed by lung function testing within the past two years or computed tomography (CT) chest scan within the past five years. Stable treatment is defined as no new or altered doses of cardiorespiratory medications (including antibiotics and oral steroids) as assessed by the study site clinician, and no hospital admissions for exacerbation of their underlying disease within the past four weeks. Participants who are actively enrolled in a PR program are excluded from participation; however past completion is permitted. No other exclusion criteria exist. A nominated carer (aged 18 years or over) per participant may optionally be enrolled. Referral of participants who are not patients of the study site clinics is permitted. Informed written consent will be obtained by an appropriately trained member of the study team.

### Intervention and data collection

**Fig. 1:**
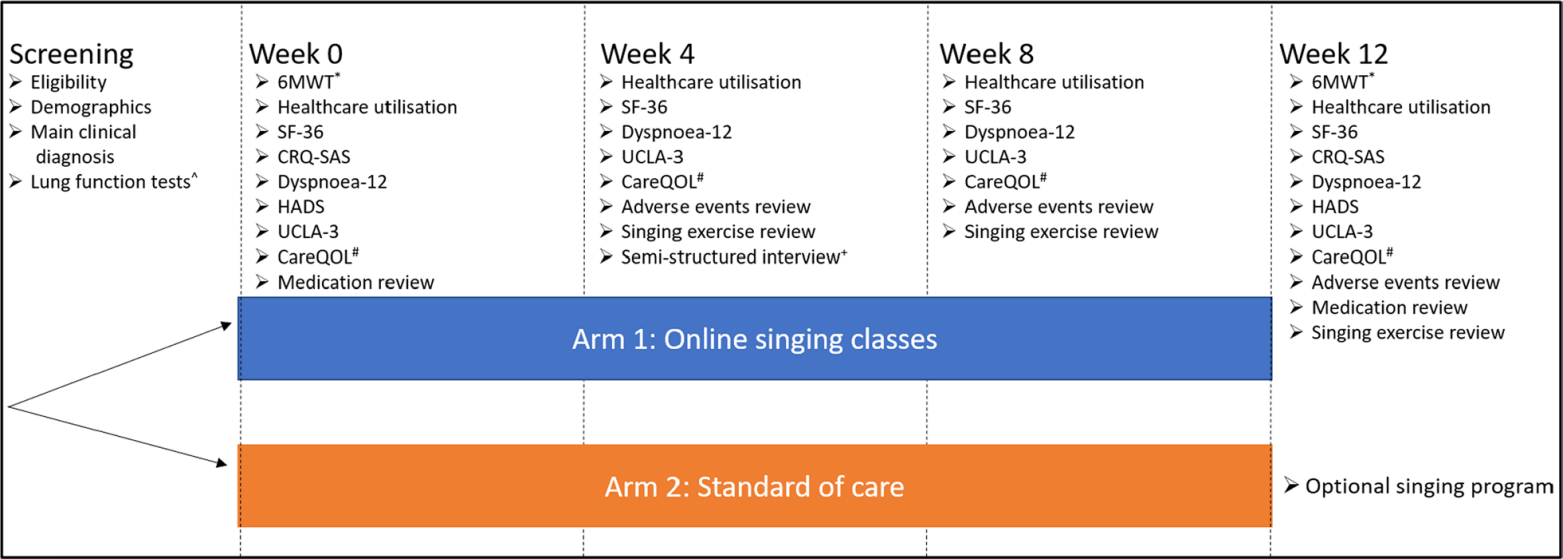
Study diagram

Participants and their carers (optional) will be enrolled for a period of 12-weeks (Fig. 1). After signing consent, participants will be randomised 1:1 to intervention or control arms with the randomisation sequence developed by the trial statistician using permuted block design (Stata statistical package [39]), and stratified by disease type (ILD vs COPD). Allocation sequence will not be visible to personnel involved in study recruitment. Participants randomised to the intervention arm will attend one 90-minute, guided, online, group singing session per week for 12 weeks. Enrollment will be on a rolling basis with a maximum of 12 patient participants and 12 carers, with up to two groups running concurrently.

The online program will be delivered via a teleconferencing platform such as Zoom. Online, group singing sessions will be conducted by a senior music therapist with expertise in singing and delivering an individualised, enjoyable singing repertoire suitable for patients with ALD (Table 1). Singing activities will focus on breathing control and will include a warm-up. Participants will be encouraged to suggest songs. During the 90 minute class, some time will be dedicated to socialisation and group connection. The exact content and structure of the online group singing sessions will be refined during phase II.

**Table 1:** SINFONIA music therapy session plan

Stage	Content	Time
Welcome and check in	•Check and provide support for technical issues •Briefly assess participants for comfort level •Identify any issues for consideration during session	5 min
Relaxation	•Relaxation exercise to support comfort and set expectations	5 min
Warm up	•Revision of techniques •Application of techniques to previous session	15 min
Main session (part I)	•Education, practice, and application of targeted breathing and singing techniques	25 min
Break	•Comfort break for bathroom or refreshments	5 min
Main session (part II)	•Application of targeted singing and breathing techniques to participants preferred repertoire	30 min
Reflection	•Reflection on content of session •Suggestions for independent practice	5 min

In addition to attending weekly, guided, online, group singing sessions, participants will be given music CDs, with repertoire and exercises from the guided group singing sessions, to utilise regularly at home. Control arm participants will receive standard care for 12 weeks, with the option to enroll in 12 weeks of guided, online, group singing at the completion of the study period (no data will be collected during this optional period). Participants and their carers will be contacted at 4-week intervals to collect study assessments. The intervention may be discontinued at any time at the participants request or if the site principal investigator deems a participant too unwell to continue. Data will be collected and stored on a REDcap server which is at least as secure as the source. Data will be accessible only by authorised members of the study team and will be stored in accordance with relevant local policies and guidelines to ensure confidentiality.

### Aims and outcomes

Phase II of the trial aims to determine the feasibility and acceptability of guided, online group singing and will collect preliminary data on effectiveness. The primary outcome of feasibility and acceptability will be assessed by the number of participants (and optional carers) that can be recruited, enrolled, and who complete the study within a six-month period (Table 2). Completion will be defined as attendance at a minimum of eight out of 12 sessions over a 12-week period for the intervention arm and completion of the 12-week assessment for the control arm.

**Table 2:** Phase II and III primary and secondary outcomes

PHASE II		
Primary outcome	Timepoint	Detail
Feasibility and acceptability	6 months	Number of participants enrolled, randomised, and who complete the program. Completion is defined as attending 8 of 12 singing sessions or completing the week 12 assessment for control arm participants
PHASE III		
Primary outcome	Timepoint	Detail
Quality of life	12 weeks	Between arm difference in SF-36
Secondary outcome	Timepoint	Detail
Anxiety and depression	12 weeks	Between arm difference in HADS
Breathlessness	12 weeks	Between arm difference in Dypnoea-12
Mastery of breathing	12 weeks	Between arm difference in mastery subdomain of CRQ-SAS
Exercise tolerance	12 weeks	Between arm difference in 6MWT
Loneliness	12 weeks	Between arm difference in UCLA-3
Healthcare utilisation	12 weeks	Between arm difference in self-reported healthcare utilisation
Carer quality of life (optional)	12 weeks	Between arm difference in carer SF-36
Patient and carer perceptions of group singing	Baseline, 4-weeks, 12-weeks	Semi-structured interview thematic analysis
Social dynamics and interactions	Throughout intervention	Ethnographic study of group classes

*SF-36* 36-Item Short Form Survey, *HADS* Hospital Anxiety and Depression Scale, *CRQ-SAS* Chronic Respiratory Disease Questionnaire—Self Administered Standardised, *6MWT* 6-Minute Walk Test, *UCLA-3* University of California Los Angeles Loneliness Scale version 3 Short Form

Phase III aims to determine whether guided, online group singing has an effect on quality of life with the primary outcome being between arm difference in quality of life (36-item Short Form Survey (SF-36)) measured at 12 weeks (Table 2) [40]. Secondary outcomes measured using between arm differences at the 12-week timepoint include effects on: anxiety and depression (Hospital Anxiety and Depression Scale (HADS)) [41], breathlessness (Dyspnoea-12) [42], breathlessness mastery (Chronic Respiratory Disease Questionnaire—Self Administered Standardised mastery subdomain (CRQ-SAS)) [43, 44], exercise tolerance (6 Minute Walk Test (6MWT)) [45], loneliness (University of California Los Angeles Loneliness Scale version 3 Short Form (UCLA-3)) [46], healthcare utilisation (self-report), carer quality of life (SF-36, optional), patient and carer perceptions of group singing (thematic analysis of semi-structured interviews) [47], and social dynamics and interactions (ethnographic observation) [48]. We hypothesise that 12 weeks of online, guided group singing will improve health-related quality of life relative to baseline compared to usual care.

Online, group singing sessions will be attended and/or recorded and reviewed by a member of the study team to conduct ethnographic analyses on the interactions between patients, carers, and singing instructors. Additionally, a purposive sample of up to 15 participants from ILD, COPD, and carer groups will be recruited for semi-structured interviews before, during, and after the intervention period to investigate perceptions of the guided, online, group singing intervention. A related ethics approval has been obtained to allow interviews with eligible participants who elect not to continue with the main study (St Vincents Hospital Melbourne HREC, LRR 111.21).

### Sample size

No formal power calculation was performed for phase II. Participant numbers will be assessed at 6-months to determine feasibility of phase III. For phase III, given a minimally clinically relevant difference in SF-36 of 10 points (SD 16) [31, 35] and assuming 80% power and 5% significance level, at least 42 participants per arm are required. To account for 40% attrition rate, at least 140 participants will be recruited. Due to the rolling nature of the singing groups, the clustering effect is assumed to be minimal, therefore no adjustment for clustering has been performed.

### Treatment allocation

Treatment allocation will be in 1:1 ratio. A randomisation sequence will be developed by the trial statistician who is not involved in recruitment using permuted block design using Stata software [39], stratified by disease type (COPD vs ILD). No trial investigators or personnel will have access to the allocation sequence.

### Safety and adverse event reporting

At each study follow up timepoint, the participants are encouraged to mention any problems since the last visit. For all randomised participants all adverse events, irrespective of causality, will be assessed and recorded as per Improving Palliative, Aged and Chronic Care through Clinical Research and Translation Trials Coordination Centre Standard Operating Procedure 5.17 Adverse Event Reporting [49]. The time period includes the time from randomisation, monthly during the intervention period and at the follow-up review. For patients who meet the eligibility criteria and then do not proceed to randomisation, any adverse events detected during that window of time, should also be reported. If an adverse event is the reason that a person does not proceed to randomisation, this should be recorded. All adverse events will be reported in summary to the reviewing HREC and site RGO and will be followed-up until: resolution; condition stabilises; event can be explained; participant is lost to follow-up; or death.

### Statistical analyses

Phase II feasibility and acceptability outcomes will be analysed descriptively. Outcomes from phase II will inform the delivery of phase III. Phase III primary and secondary outcomes will be analysed on intention-to-treat basis using mixed effect linear regression. Participants will be entered as random coefficients, while time, treatment arm and an interaction between them will be entered as fixed terms. Residuals will be visually inspected and if the model is of poor fit, the outcome will be transformed using natural logarithms. The primary outcome will be expressed as the difference in SF-36 at 12 weeks with 95% confidence intervals. If the model remains of poor fit despite the outcome transformation, then mixed effect ordinal logistic model will be used and results will be expressed as odds ratios with 95% confidence intervals. Additional per protocol analysis will be performed using a subgroup of participants who have completed the last visit and have completed at least 8 sessions over 12 weeks.

Multiple exploratory subgroup analyses will be performed, including: sex (female vs male), age group (60 years, 60–70 years, or > 70 years), disease severity (mild-moderate vs severe), disease type (COPD vs ILD), prior psychological disease (present vs absent), breathlessness (mMRC of 2 vs 3 vs 4), and previous pulmonary rehabilitation (yes vs no). Subgroup analysis will be performed by including an interaction term between subgroup and treatment arm in the initial model. Additional analysis will involve the associations between change in the outcome and compliance to intervention and presence of carer in group sessions. These will be performed using linear regression. Outcomes are collected at multiple time-points throughout the study to allow for better missing data imputation, which will be performed within the mixed effects model (using maximum likelihood estimation).

Semi-structured interviewing will continue until sufficient information power is reached [50]. Given the narrow scope of the qualitative analyses, it is anticipated that sufficient information will be attained with a maximum of 15 COPD patients, 15 ILD patients and 15 carers. Each interview will be transcribed verbatim and thematically analysed for perceived patient and carer preferences, barriers, and opportunities based on the approach described by Braun and Clarke [47]. A purposive selection of session recordings will be analysed for patient experiences, actions, behaviours, and responses based on the approach described by Goodson and Vassar [48]. These qualitative outcomes will supplement the quantitative primary and secondary outcomes from phase III.

### Reporting results

This protocol paper has been prepared in accordance with Standard Protocol Items: Recommendations for Interventional Trials (SPIRIT) guidelines [51, 52]. All results will be reported according to the Consolidated Standards of Reporting Trials (CONSORT) statement for reporting randomised trials [53]. A participant flow diagram will include baseline characteristics, distribution of participants between arms, and number of participants with missing data. Baseline characteristics will be presented by arm using median (with interquartile range) for non-parametric data, or mean (with standard deviation) for normally distributed continuous variables; and as frequencies (percentages) for categorical variables. There will be no adjustment for multiple secondary outcomes, however, the results of secondary outcomes will be interpreted in light of multiple comparisons and focus will be given to their clinical significance.

## Discussion

The SINFONIA study aims to determine the feasibility, acceptability, and effectiveness of guided online group singing as an intervention to improve quality of life in people with COPD and ILD patients and significant breathlessness, and their carers. This study is the first of its kind in Australia and to our knowledge, the first to deliver a singing intervention program to this patient group entirely online.

Some benefits of group singing in patients with ALD have been attributed to the social support and connection with other people with a similar condition that is incidentally formed via regular participation in a group activity. Online delivery represents a major hurdle in delivery of this intervention and it is unclear whether online group singing can facilitate comparable social connection opportunities and thus overall benefits. A similar singing intervention has been delivered entirely online over six weeks to participants experiencing persistent symptoms following COVID-19 infection (long COVID-19) in the UK, with singing resulting in an improvement in the mental health component of the SF-36 when compared with usual care [54]. These results are promising, however the mean age of long COVID-19 patients in that study is considerably lower than that of other ALD patients participating in face-to-face group singing (49 vs 71 years) [32]. Digital literacy is likely to be much greater amongst younger cohorts, indeed 70% of long COVID-19 participants anticipated no barriers to participation in an online intervention at baseline [54]. The same trend does not appear to hold true for other ALD patients. One prior study transitioned from face-to-face delivery to online five weeks into a 12-week intervention and reported a decline in attendance from 90 to 53% [33]. Participants in that study cited online access and digital literacy as barriers to participation. Semi-structured interviews and ethnographic analyses conducted in the current study aim to identify whether these barriers are present in the current cohort. Additionally, separate ethics approval will allow interviews with eligible participants who elect not to continue with the study and may help identify strategies to improve access for these groups.

A major confounding variable across previous respiratory, group singing studies is whether the participant in the singing program had undertaken PR or not at the same time as participating in guided, group singing activities. Two previous singing interventions recruited participants directly from PR groups [28, 55] however, this makes it difficult to separate benefits from singing programs from those arising from recent PR. Later trials have specifically targeted patients with no recent PR involvement [32] in an attempt to better control this variable. Aside from the clinical benefits of PR, patients from that cohort have a proven ability to engage with regular, group health interventions that occur over several weeks. A current trial from the “Sing Your Lungs Out” (SYLO) group in New Zealand is specifically targeting people who have refused or not been offered PR, however, that study is yet to return results [28]. Our trial excludes patients currently enrolled in PR, but permits prior attendance, in an effort to best replicate real-world application of guided online group singing as a community-based intervention, which can potentially augment and prolong the known benefits of PR, and additionally, may appeal to patients who are otherwise reluctant to engage in PR.

Of the two major implementations of guided, group singing in respiratory health, SYLO and Singing for Lung Health (SLH, UK), both groups have maintained high levels of ongoing participation beyond the initial intervention period, reflective of the perceived value to participants. SYLO reported an 85% attendance rate throughout the intervention and participants continued to regularly meet 18 months after inception [36, 56]. Similarly, SLH is now an expansive network of affiliated and independent groups across the UK. Whilst the demand for guided, group singing programs for people with COPD and other lung conditions is evident, there is no clear pipeline of funding or ongoing support to maintain these groups beyond a trial period. Both SYLO and SLH have adopted a model of encouraging groups to become self-sufficient and both largely rely on volunteer efforts and donations to remain viable. This donation-driven model of funding raises issues of accessibility and equity and as such, healthcare economics analyses are essential to determine the feasibility of guided singing groups as a funded health intervention. A service evaluation of SLH retrospectively assessed healthcare utilisation of 228 participants with COPD following 12 weeks of guided, group singing and reported a reduction in general practitioner visits and hospital admissions, though this data was derived from self-reported recall and had no control group [27]. The more robust measure of hospital admissions from patient medical record audit data were captured for SYLO participants, with no changes in admission frequency identified following one year of attending the guided, group singing program. However, again there was no control group, with instead data compared to historical baseline admission data for participants [28]. Our current study utilises self-reported healthcare utilisation data collected on a 4-weekly basis throughout the trial period, with a standard care control arm comparator to determine any unscheduled healthcare utilisation benefits in the Australian context.

## Conclusion

Patients with COPD and ILD live with a severe and prolonged symptom burden. Current therapeutic interventions are known to be effective but are often limited in uptake due to a range of referral and participation barriers. Online guided, group singing is a low-risk, low-cost intervention which can be delivered in the community that may have physiological and/or psychosocial benefits to patients with COPD or ILD. The SINFONIA study is a phase II/III clinical trial of online guided, group singing as an intervention to improve quality of life in patients with COPD or ILD and breathlessness.

## Data Availability

Not applicable.

## Abbreviations

6MWT: 6-Minute walk test
ALD: Advanced lung disease
CONSORT: Consolidated Standards of Reporting Trials
COPD: Chronic obstructive pulmonary disease
CRQ-SAS: Chronic Respiratory Disease Questionnaire—Self Administered Standardised
CT: Computed tomography
HADS: Hospital Anxiety and Depression Scale
ILD: Interstitial lung disease
mMRC: Modified medical research council dyspnoea score
PR: Pulmonary rehabilitation
SF-36: 36-Item Short Form Survey
SINFONIA: A clinical trial examining the benefits of **S**ing**IN**g **FO**r breathing in COPD a**N**d **I**LD p**A**tients
SLH: Singing for Lung Health (UK singing intervention)
SPIRIT: Standard Protocol Items: Recommendations for Interventional Trials
SYLO: Sing Your Lungs Out (New Zealand intervention)
UCLA-3: University of California Los Angeles Loneliness Scale version 3 Short Form
